# Anti-MDA5 antibody positive dermatomyositis complicated by interstitial lung disease with an improved outcome: A case report

**DOI:** 10.1097/MD.0000000000042536

**Published:** 2025-05-23

**Authors:** Qian Jiang, Yongji Liu, Xiuhui Chai, Xiaoping Yang, Bin Yin, Yong Wang, Lei Xu, Wen Jiang, Maoqing Xu, Wenqing Jiang

**Affiliations:** a Department of PCCM2, Qingdao Haici Hospital Affiliated to Qingdao University, Qingdao, Shandong, China; b Deparment of Internal Medicine, Qingdao Public Health Clinical Center, Qingdao, Shandong, China.

**Keywords:** case report, dermatomyositis, interstitial lung disease, MDA5

## Abstract

**Rationale:**

Anti-melanoma differentiation-associated gene 5 (MDA5) antibody positive dermatomyositis (MDA5-DM) is an autoimmune disease, characterized by a particular skin ulcer and rapidly progressive interstitial lung disease (RP-ILD). This case highlights diagnostic challenges of this disease and underscores the importance of early recognition and timely intervention.

**Patient concerns:**

A 59-year-old woman presented to the hospital with systemic myalgia, fatigue, and anorexia. Following admission she developed recurrent fever. Computed tomography of the chest and bronchoalveolar lavage fluid analysis revealed severe pulmonary infections. Despite 2 weeks of antimicrobial therapy yielding radiographic improvement, her dermatomyositis symptoms worsened.

**Diagnoses:**

Immune profiling and myositis-specific antibody testing confirmed the diagnosis of MDA5-DM-ILD.

**Interventions:**

The following treatment included methylprednisolone combined with immunosuppressants (tofacitinib, tacrolimus, tocilizumab, mycophenolate mofetil). Subsequent computed tomography imaging demonstrated cavity lesions and micronodules, with lung biopsy confirming fungal and viral coinfections. Therapy was adjusted to sustained methylprednisolone (22 mg/d), tacrolimus (0.5 g/d), targeted antimicrobials (ganciclovir, voriconazole) and prophylactic use of sulfamethoxazole complex.

**Outcomes:**

At discharge, the patient achieved clinical stability with resolved fever, normalized oxygen saturation and improved myalgia, though mild epigastric discomfort persisted.

**Lessons:**

MDA5-DM-ILD poses challenges for clinicians due to its diagnostic difficulty, rapid progression and secondary infection risks. Early immunosuppression combined with infection prevention is the key to a successful treatment of this disease.

## 1. Introduction

Anti-melanoma differentiation-associated gene 5 antibody positive dermatomyositis (MDA5-DM) is an autoimmune disorder characterized by cutaneous ulcers and rapidly progressive interstitial lung disease (RP-ILD). Most patients develop DM-associated ILD within months of disease onset. We herein introduce the case of a 59-year-old woman who presented with systemic myalgia, fatigue and anorexia, who was ultimately diagnosed with MDA5-DM. Following hospitalization, the patient exhibited recurrent febrile episodes. Imaging and microbiological analyses revealed severe pulmonary infections: chest computed tomography (CT) demonstrated bilateral ground glass opacities, while bronchoalveolar lavage fluid cultures identified polymicrobial pathogens. Although 2 weeks of antimicrobial therapy yielded radiographic improvement, her DM symptoms progressed markedly.

Definitive diagnosis was established through immunoserological profiling, demonstrating positivity for anti-MDA5 and anti-Ro52 antibodies. Combination of immunosuppressive therapy was initiated, including methylprednisolone pulse therapy (80 mg/d) with several immunosuppressants (IS; tofacitinib, tacrolimus, tocilizumab, and mycophenolate mofetil [MMF]), resulting in partial clinical improvement. Subsequent CT imaging at 1 month follow-up revealed emerging cavitary lesions and diffuse micronodules. Percutaneous lung biopsy confirmed fungal and viral coinfections, necessitating therapeutic escalation to voriconazole, ganciclovir and sulfamethoxazole complex (SMZ-co). Maintenance therapy was tapered to oral methylprednisolone (22 mg/d) and tacrolimus (0.5 mg/d). At discharge, the patient achieved clinical stability with resolved fever, normalized oxygen saturation (SpO₂ > 95% on room air) and attenuated myalgia, though mild epigastric discomfort persisted.

This case underscores the imperative for meticulous clinical evaluation in MDA5-DM management. Diagnostic delays due to atypical early presentations and therapeutic complexities arising from infection-immunosuppression interplay highlight the necessity for early serological testing, aggressive immunomodulation, and proactive surveillance of opportunistic infections.

## 2. Case description

### 2.1. On admission

A 59-year-old female patient was initially admitted to the Department of Spine Surgery with an 8-year history of chronic lower back pain and generalized myalgia. Previous therapeutic interventions, including traditional Chinese medicine physiotherapy and intravenous analgesics, provided minimal relief. The patient subsequently developed progressive gastrointestinal symptoms (reduced oral intake, belching) and recurrent febrile episodes (maximum temperature 38.9°C). Laboratory investigations revealed as follows: leukopenia (white blood cell 3.21 × 10⁹/L) with lymphocytopenia (lymphocyte 0.50 × 10⁹/L), elevated lactate dehydrogenase (lactic dehydrogenase 288.3 U/L) and weakly positive influenza A virus immunoglobinM antibodies. Chest CT demonstrated bilateral interstitial infiltrates, pleural thickening and minimal pleural effusion (Fig. 3A), while abdominal CT showed no significant pathology. Initial management with intravenous moxifloxacin (400 mg q24h) and antipyretic agents was given. Due to progressive respiratory compromise, the patient was transferred to the Pneumology Department on hospital day 4 for further treatment of suspected severe pulmonary infection.

On physical examination, the patient exhibited persistent low-grade fever (38°C), generalized asthenia, anorexia, epigastric pain and exertional dyspnea. Cardiopulmonary auscultation revealed clear breath sounds without obvious rales. Neuromuscular assessment demonstrated preserved muscle tone with asymmetric weakness. Cutaneous examination showed erythematous discoloration over malar regions, periocular areas, digits and chest wall, without definitive rash formation.

Based on preliminary findings, the following regimen was initiated: Peramivir 0.3 g via intravenous drip (IVGTT) daily × 5 days for antiviral treatment; Methylprednisolone 40 mg IVGTT daily × 5 days for immunomodulation; Moxifloxacin 400 mg IVGTT daily for antimicrobial treatment (β-lactam allergy documented); Pantoprazole 40 mg IVGTT daily + Mosapride 5 mg taken orally 3 times a day to relief gastric symptoms; High-flow nasal cannula (FiO₂ 35%, flow 30 L/min) for respiratory support.

**Figure 1.**
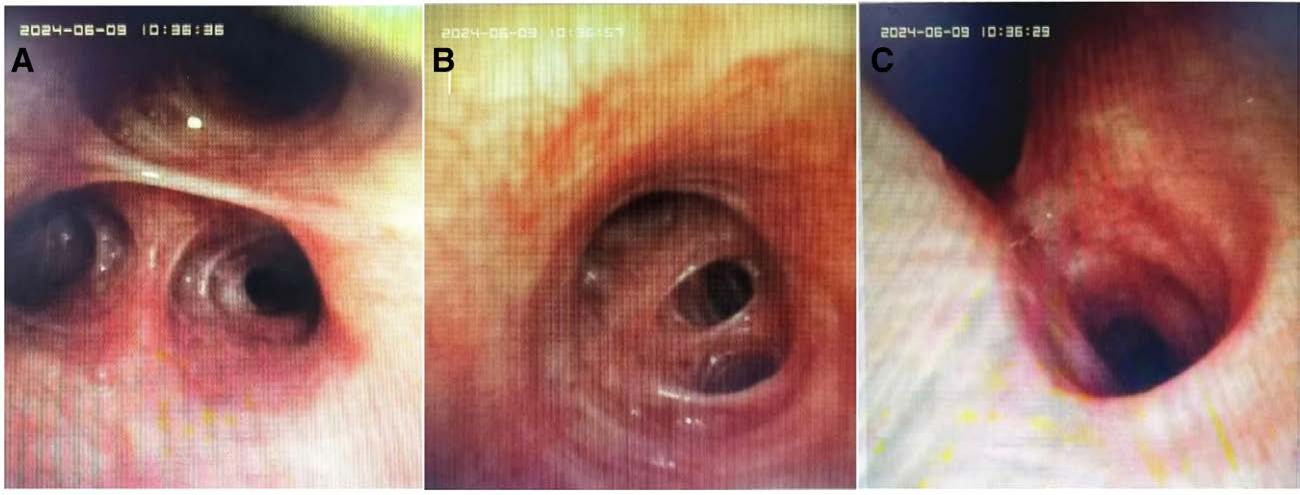
Features under bronchoscopy. (A) Righter upper lobe. (B) Opening of the right lower lobe. (C) The carina.

Arterial blood gas analysis revealing hypoxemia (PaO₂ 49 mm Hg, SpO₂ 86.2%) and elevated D-dimer prompted CT angiography, which excluded thromboembolic events. Esophagogastroduodenoscopy confirmed chronic superficial gastritis with benign polyps. By day 5, partial symptomatic improvement was noted (SpO₂ 92% on room air), though low-grade fever (37.5–38°C) persisted. On laboratory reexamination, cytopenias (white blood cell 2.93 × 10⁹/L) and transaminitis (aspartate transaminase: 145.6 U/L, alanine transaminase: 168.2 U/L) occurred. Further immunological evaluation turned out as follows: hyperferritinemia: 1063 ng/mL; autoantibodies: anti-Ro52 (+), antinuclear antibodies 1:100 speckled pattern; pulmonary function showing forced vital capacity 86.5%, diffusing capacity of the lung for carbon monoxide 48.8% predicted. Bronchoscopic evaluation (Fig. 1) with bronchoalveolar lavage metagenomic sequencing identified polymicrobial flora: *Streptococcus constellatus, Acinetobacter baumannii complex, Saccharomyces cerevisiae and Candida albicans*.

**Figure 2.**
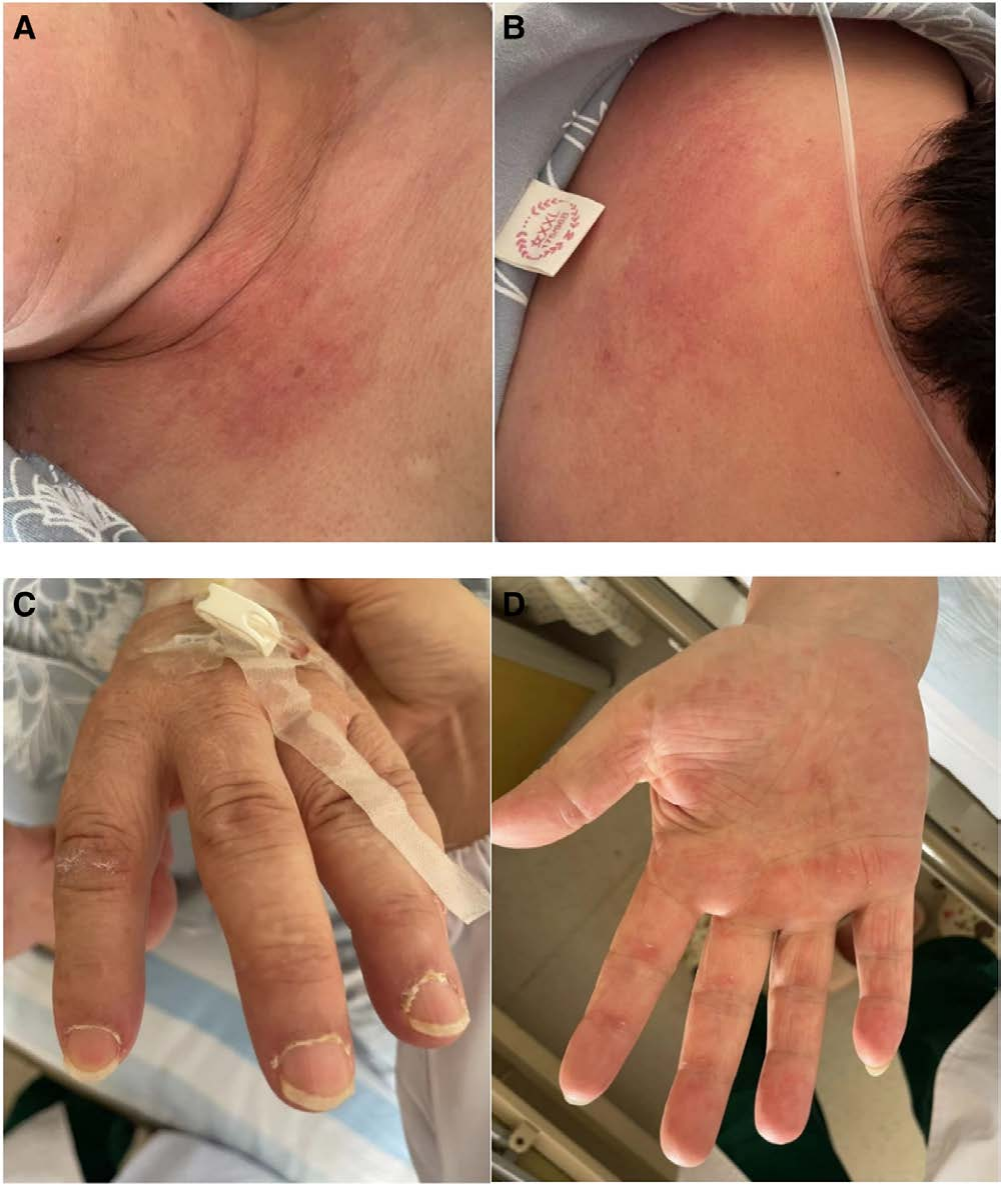
Skin features. (A, B) Red rashes on the anterior chest and back and shoulders, conforming to the V-neck sign and shawl sign. (C, D) Skin desquamation on the extended surfaces of both hands near the knuckles and elbows, keratosis on the index fingers of both hands, conforming to the Gottron sign and mechanic’s hands.

Rheumatological consultation revealed pathognomonic cutaneous manifestations: Gottron papules over metacarpophalangeal joints, mechanic’s hands with palmar hyperkeratosis, V-neck and shawl sign (Fig. 2). Serological findings of anti-MDA5 (+++) and anti-Ro52 (++) antibodies established the diagnosis of MDA5-DM-ILD.

The following therapeutic interventions were implemented. SMZ-co 80 mg twice a day (BID) was given for Pneumocystis jirovecii pneumonia (PJP) prophylaxis. For immunosuppressive regimen, methylprednisolone pulse therapy was started by 80 mg IVGTT daily, tapered to 40 mg (day 6–10), 32 mg (day 11–20), and maintained at 24 mg (day 21 onward). Tocilizumab 8 mg/kg IVGTT every 2 weeks was initiated for refractory hyperferritinemia (peak 1750 ng/mL). Tofacitinib was started but discontinued due to cardiotoxicity (persistent chest discomfort, T-wave inversion and troponin-I elevating to 0.576 ng/mL). Tacrolimus was switched to MMF 750 mg BID following hyperkalemia (serum K⁺ 6.1 mmol/L). Immunoglobulin IVGTT 10 g × 3 consecutive days per 15-day cycle was given for adjunctive therapies. Fluconazole was escalated to voriconazole for antifungal treatment.

**Figure 3.**
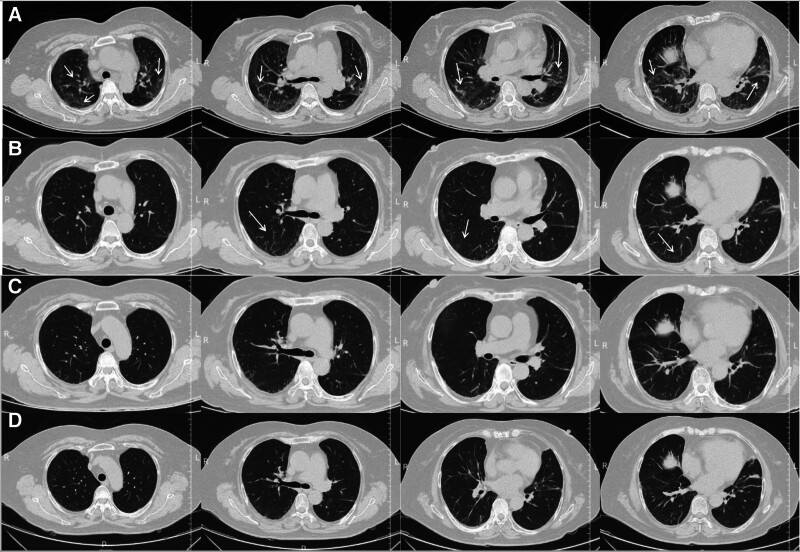
Chest CT changes. (A) Upon admission, chest CT showed interstitial change where linear and patchy high-density shadows were seen subpleurally, bilateral pleural hypertrophy and mild pleural effusion. (Arrows). (B) Two weeks after anti-infection therapy, linear and patchy shadows reduced. (Arrows). (C) One month after admission. (D) Last CT reexamination of 1st hospitalization. CT = Computed tomography.

Serial chest CT evaluations (Fig. 3A–D) demonstrated gradual resolution of ground glass opacities and interlobular septal thickening over 4 weeks, consistents with treatment-responsive ILD. Upon discharge (hospital day 28), the patient exhibited hemodynamic stability (afebrile > 72 hours, SpO₂ 96% on room air) and improved symptoms with only occasional stomachache.

### 2.2. Post discharge

**Figure 4.**
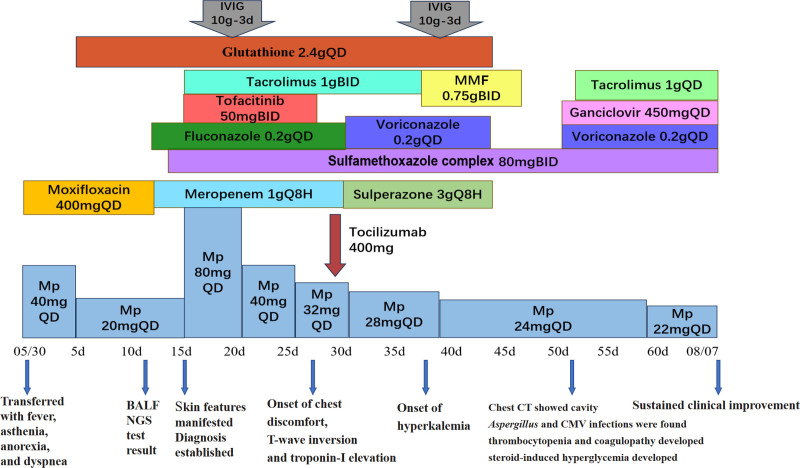
Course of diseases and medication adjustment. BALF = bronchoalveolar lavage fluid, BID = twice a day, CMV = cytomegalovirus, CT= computed tomography, IVIG = intravenous gamma globulin, MMF = mycophenolate mofetil, Mp = methylprednisolone, NGS = next-generation sequencing, QD = once a day.

At 2-month follow-up, the patient remained free of cutaneous manifestations or respiratory distress. However, chest CT revealed newly onset of right upper lobe cavitary nodulation, scattered micronodules, and mild pericardial effusion. CT-guided lung biopsy with metagenomic next-generation sequencing identified *Pseudomonas putida, Stenotrophomonas maltophilia, Nocardia farcinica, Aspergillus terreus and Human cytomegalovirus* (*CMV*). Antimicrobial therapy was then optimized to voriconazole combining with ganciclovir. Immunosuppression was adjusted according to therapeutic drug monitoring: MMF discontinued for 5 days prior to transitioning to tacrolimus 0.1 g daily with methylprednisolone tapered to 22 mg daily (Fig. 4).

The patient later developed thrombocytopenia and coagulopathy possibly attributed to CMV reactivation and sepsis. After receiving ganciclovir, the platelet level and coagulation returned to normal. She also developed steroid-induced hyperglycemia, which was then managed with glucose control: Metformin 0.5 g BID, Acarbose 50 mg 3 times a day and short-acting insulin.

### 2.3. Follow-up and outcomes

The patient was discharged 20 days following secondary admission with sustained clinical improvement. Maintenance therapy with intravenous immunoglobulin (IVIG) 10 g monthly was continued. Final outpatient evaluation demonstrated improved laboratory findings. The patient and family expressed gratitude for the diagnostic diligence and coordinated care, enabling therapeutic adherence throughout the treatment course. By recent online contact, the patient said that she was in a stable condition with symptoms limited to intermittent fatigue and mild appetite reduction.

## 3. Discussion

MDA5-DM is a subset of DM characterized by anti-MDA5 autoantibodies and distinct clinical manifestations, including skin ulcers and RP-ILD. Diagnostic Criteria (Europwan Neuromuscular Centre, ENMC, 2018) includes presence of Gottron sign (papules on knuckles) or heliotrope rash (purple eyelids) combined with serum anti-MDA5 antibodies.^[1]^

The heterogeneity in clinical presentation of MDA5-DM, particularly the absence of classic cutaneous or muscular features at disease onset, poses significant diagnostic challenges and requires elaborate clinical investigation. In some cases, the absence of both cutaneous and muscle manifestations may culminate in a delayed diagnosis of RP-ILD, with fatal outcomes within weeks of symptom onset in up to 50% of cases.^[2,3]^ In our case, classic rashes (e.g., Gottron’s sign, heliotrope) appeared nearly 10 days post-admission, resulting in a delay of diagnosis to a certain extent. While there has been no studies of such aspect, it is thought that shin changes might correlate with disease activity (e.g., ILD progression, ferritin levels).

The onset of MDA5-DM involves genetic predisposition, environmental triggers and autoimmune dysregulation, where the type I interferon (IFN-I) pathway, anti-MDA5 IgG and various immune cells may all participate.^[4]^ Recent publications have suggested that over-activation of IFN-I signaling may drive vasculopathy, contributing to both cutaneous and pulmonary lesions.^[2]^ Viral infection is thought to be a possible trigger for MDA5-DM. The MDA5 antibody has been tested in some COVID-19 patients and is recognized as an RNA sensor and a key pattern recognition receptor for the SARS-CoV-2 virus.^[5]^ Furthermore, MDA5-DM patients have similar expression levels to those of COVID-19, suggesting the likelihood of viral infection inducing MDA5-DM onset.^[6,7]^ In our case, the patient had a trace of influenza A virus infection on admission, but prolonged immunosuppression use likely enabled overgrowth of various pathogens. This seems to reflect the paradox of MDA5-DM therapy where aggressive immunosuppression is needed to halt RP-ILD but also risks unleashing pathogens.

A high burden of opportunistic lung infection is already the most common complication of MDA5-DM. Nearly 50% of patients develop infections (CMV, fungal, PJP or bacteria), often 3 months post-diagnosis as immunosuppression intensifies. The reported detection rate of PJP in bronchoalveolar lavage fluid next-generation sequencing of MDA5-DM patients is as high as 48.4%.^[8]^ Thus prophylactic use of SMZ-co at the time of diagnosis is highly recommended. For our patient, 1st-line therapy was started from beginning with high-dose glucocorticoids and calcineurin inhibitors (tacrolimus), as well as Janus kinase inhibitors (tofacitinib) to mitigate IFN-I-driven lung injury and intravenous immunoglobulin to modulate autoantibodies without further immunosuppression. Later development of treatment-related adverse events such as hyperkalemia and myocardial enzyme abnormality prompted dose adjustments and gradual tapering of glucocorticoids, leading to an eventually balance between efficacy and safety.

Thus for MDA5-DM management, a precision medicine approach, dynamic and personalized regimens, as well as vigilant infection prevention are all of great importance.

In our patient’s myositis antibody spectrum, both MDA5 and Ro-52 antibodies were positive.

Ro-52, a key immunomodulatory protein, regulates apoptosis, autophagy, viral infection and antiviral immune responses, and is linked to systemic lupus erythematosus, Sjogren’s syndrome, mixed connective tissue disease and DM. Its positivity is considered a poor prognostic marker for inflammatory myopathy.^[9]^ Studies show that double positivity for MDA5 and Ro-52 antibodies increases the risk of RIP-LD fivefold compared to single MDA5 positivity.^[10]^ Additionally, double-positive patients exhibit higher lactic dehydrogenase, higher ferritin, lower albumin levels, lymphopenia and hypoxemia, leading to worse prognosis and higher mortality.

Generally recognized effective treatment strategies for MDA5-DM are still unclear. Glucocorticoids remain the 1st-line therapy, often combined with IS for patients with severe rash or ILD. Calcineurin inhibitors like tacrolimus and cyclosporine are widely used. One regimen promoted by Japanese researchers of high-dose glucocorticoids, tacrolimus and cyclophosphamide has shown efficacy.^[11]^ Other clinical studies have also highlighted that using IS as early as possible after diagnosis may improve disease prognosis.^[12]^ However, these treatments are associated with significant adverse events, particularly infections. Bacterial, viral and fungal infections, including CMV reactivation and PJP, have all been reported.^[13]^ In our case, the patient developed a CMV lung infection shortly after initiating multiple IS, prompting the immediate commencement of antiviral therapy.

Small-molecule targeted drugs and biological agents show promise in treating MDA5-DM. Studies suggest that early initiation of the Janus kinase inhibitor tofacitinib can be effective.^[14]^ The Janus kinase-signal transducer and activator of transcription signaling pathway, activated by IFNs, promotes the transcription of IFN-stimulated genes, thus including MDA5. Tofacitinib can inhibit this pathway, reducing MDA5 expression and activation. However, the benefit-risk balance of tofacitinib varies among patients and requires further investigation. In our case, the patient developed chest tightness and cardiac abnormalities after starting tofacitinib, necessitating discontinuation of the drug. While some studies report major adverse cardiovascular events associated with tofacitinib,^[15]^ these findings remain inconclusive.

## Acknowledgments

The authors thank Ms. Xu for her rheumatology expertise for this case report. Ms. Xu has permitted her name to be mentioned.

## Author contributions

**Conceptualization:** Qian Jiang.

**Resources:** Yongji Liu, Xiuhui Chai, Xiaoping Yang, Bin Yin, Yong Wang, Lei Xu, Wen Jiang, Maoqing Xu, Wenqing Jiang.

**Writing – review & editing:** Qian Jiang.

**Writing – original draft:** Qian Jiang.
